# Factors that Influence the Emotional Impact of Memory Problems in Older Adults: A Mixed-Methods Study

**DOI:** 10.1093/geroni/igab046.2602

**Published:** 2021-12-17

**Authors:** Emily Bratlee-Whitaker, Nikki Hill, Jacqueline Mogle, Rachel Wion, Caroline Madrigal, Sakshi Bhargava

**Affiliations:** 1 Penn State University, University Park, Pennsylvania, United States; 2 Indiana University, Indiana University, Indiana, United States; 3 Providence VA Medical Center, Providence VA Medical Center, Rhode Island, United States

## Abstract

Older adults’ experiences with memory problems may be an important indicator of current and future well-being; however, these experiences and their impacts are poorly characterized, particularly in those with co-occurring affective symptoms. The purpose of this mixed-methods study was to examine how the experience of memory problems influences emotional well-being in older adults without dementia, and whether this differs based on cognitive status and current depressive symptoms or anxiety symptoms. A convergent parallel mixed methods design was used in which quantitative and qualitative data were collected simultaneously, analyzed separately, and then integrated to determine how participants’ experiences differed. Community-dwelling older adults (n=49, Mage = 74.5, 63% female) without severe cognitive impairment completed study questionnaires and two individual, semi-structured interviews. Five themes were identified that described the influence of memory problems on emotional well-being: Evoking Emotions, Fearing Future, Undermining Self, Normalizing Problems, and Adjusting Thinking. The extent to which memory problems impacted emotional well-being depended on multiple factors including current affective symptoms (primarily anxiety), characteristics of the experience (such as judgments of its importance), as well as personal experience with dementia. Notably, there were no thematic differences in the emotional impact of memory problems between older adults with normal cognition and those with evidence of mild cognitive impairment. Our findings suggest that thorough assessment of reports of memory problems, regardless of cognitive testing outcomes, should consider co-occurring subsyndromal affective disorders as well as older adults’ evaluations of how memory problems influence their daily lives and well-being.

